# Vitamin D Receptor Genetic Variations May Associate with the Risk of Developing Late Fracture-Related Infection in the Chinese Han Population

**DOI:** 10.1155/2022/9025354

**Published:** 2022-02-10

**Authors:** Xing-qi Zhao, Kun Chen, Hao-yang Wan, Si-ying He, Han-jun Qin, Bin Yu, Nan Jiang

**Affiliations:** ^1^Division of Orthopaedics & Traumatology, Department of Orthopaedics, Southern Medical University Nanfang Hospital, Guangzhou 510515, China; ^2^Guangdong Provincial Key Laboratory of Bone and Cartilage Regenerative Medicine, Southern Medical University Nanfang Hospital, Guangzhou 510515, China

## Abstract

Variations in the vitamin D receptor (*VDR*) gene are related to several inflammatory disorders. However, the potential links between such alternations and the risk of developing late fracture-related infection (FRI) remain unclear. This study investigated associations between genetic variations in the *VDR* and susceptibility to late FRI in the Chinese Han population. Between January 2016 and December 2019, 336 patients with late FRI and 368 healthy controls were genotyped six *VDR* genetic variations, including *ApaI* (rs7975232), *BsmI* (rs1544410), *FokI* (rs2228570), *TaqI* (rs731236), *GATA* (rs4516035), and *Cdx-2* (rs11568820). Significant associations were observed between rs7975232 and FRI susceptibility in the recessive (*P* = 0.019, OR = 0.530, 95% CI 0.310–0.906) model. Patients with AA genotype had a relatively higher level of serological vitamin D (20.6 vs. 20.3 vs. 17.9 ng/ml) (*P* = 0.021) than those of AC and CC genotypes. Although no statistical differences were observed, potential correlations may exist between rs1544410 (dominant model: *P* = 0.079, OR = 0.634), rs2228570 (dominant model: *P* = 0.055, OR = 0.699), and rs4516035 (dominant model: *P* = 0.065, OR = 1.768) and the risk of FRI development. In the Chinese cohort, *ApaI* was associated with a decreased risk of developing FRI, and patients with the AA genotype had a higher vitamin D level. Further studies are required to assess the role of genetic variations in *BsmI*, *FokI*, and *GATA* in the pathogenesis of late FRI.

## 1. Introduction

Fracture-related infection (FRI), one of the most frequent types of bone infection, refers to osseous infection with or without the surrounding soft tissue infection following trauma and/or orthopaedic surgery [1], with infections secondary to internal fixation and open fracture as the primary types. The average incidence of FRI was approximately 5%, with 1% to 2% after closed fractures, which exceeding 30% following open fractures [2]. Currently, successful management of FRI poses substantial challenges for orthopaedic surgeons, which is primarily attributed to its characteristics of high heterogeneity, i.e., despite the same disorder, clinical efficacy varies among patients, and is affected by factors such as infection site and duration, pathogen type and virulence, immune status, and treatment strategy. Nonetheless, the overall efficacy remains unsatisfactory, posing great pressures on the patients, not only physically but also psychologically [3, 4] and economically [5, 6]. Even in patients with long-term skin ulcers and sinus drainage, malignant transformation of squamous cell carcinoma is not rare [7]. Therefore, reducing the incidence of FRI is of great personal and social significance and needs to be built on a comprehensive understanding of FRI pathogenesis.

The pathogenesis of FRI is complex, which associates with both extrinsic and intrinsic factors. However, most previous studies have reported FRI pathogenesis from the perspective of environmental factors, which are largely controllable. Recently, growing evidence has shown that genetic predisposition also plays an important role in FRI development, with single nucleotide variation (SNV) as a representative. Several SNV sites have been found to be associated with the risk of FRI development, such as rs689466 (cyclooxygenase-2, *COX-2* gene) [8]; rs16944, rs2234663, rs1143627, rs4251961, and rs1800796 (interleukin, *IL* genes) [1, 9]; and rs2430561 (interferon-*γ*, *IFN-γ* gene), demonstrating that as a host factor, SNV is also involved in developing FRI.

Vitamin D participates in several biological processes, such as bone metabolism, regulation of cell proliferation and differentiation, and modulation of the immune response. Vitamin D mediates its function by binding to the vitamin D receptor (VDR), encoded by the *VDR* gene, and controls the synthesis of different proteins [10]. The *VDR* gene is highly polymorphic, significantly influencing the functioning of the VDR protein and thus may influence the occurrence of disorders. The most frequently reported *VDR* genetic variations include *ApaI* (rs7975232), *BsmI* (rs1544410), *FokI* (rs2228570), *TaqI* (rs731236), *GATA* (rs4516035), and *Cdx-2* (rs11568820). Previous studies have indicated that these SNVs are associated with the risk of developing several inflammatory disorders, such as tuberculosis [11], chronic periodontitis [12], paediatric urinary tract infection [13], and *Helicobacter pylori* infection [14]. A previous study found that *TaqI* (rs731236) and *FokI* (rs2228570) increased the risk of developing chronic osteomyelitis (COM) in the Chinese population [15]. However, both FRI and non-FRI patients were included as an entire entity for analysis, resulting in heterogeneity. Additionally, the sample size of the participants was limited. Moreover, potential relationships between different genotypes and serum vitamin D levels were not explored. Thus, the effects of genotype on vitamin D levels in patients with FRI remain unclear.

To address the above-mentioned drawbacks and questions, we investigated potential associations between *VDR* gene variations (*ApaI*, *BsmI*, *FokI*, *TaqI*, *GATA*, and *Cdx-2*) and the risk of late extremity FRI development in the Chinese Han population.

## 2. Materials and Methods

### 2.1. Definition, Inclusion and Exclusion Criteria, and Study Registration

The present study was designed as a case-control investigation, with comparisons conducted between patients with FRI and healthy controls. Late FRI is defined as bone infection with or without surrounding soft tissue infection following open fractures or internal fixation for closed fractures, with an infection duration exceeding 10 weeks [16, 17]. FRI was established based on any of the four confirmatory criteria: wound breakdown to the bone or implant, sinus or fistula connecting the bone or implant, positive pathogen culture, and histological test outcomes [18]. Participants included in the patient group were those who had been diagnosed with FRI at the Southern Medical University Nanfang Hospital between 1 January 2016 and 31 December 2019. Patients with anther aetiologies of COM (haematogenous spread and diabetic foot infection) were receiving vitamin D supplementation and diet were excluded. Eligible participants in the control group were defined as healthy after thorough examinations at the hospital. All the included participants signed the informed consent form, and this study was conducted in accordance with the tenets of the 1964 Helsinki declaration. This study was approved by the Medical Ethics Committee of the hospital (NFEC-2019-087). The protocol of this study was registered in the Chinese Clinical Trial Registry (ChiCTR1900022186).

### 2.2. DNA Extraction and SNV Genotype

Ethylene diamine tetraacetic acid (EDTA) peripheral blood samples (5 ml each) were collected and stored at –80°C. Then, the genomic DNA of each sample was extracted from the peripheral blood leukocytes according to the instructions of the Flexi Gene-DNA Kit (Qiagen, Valencia, CA). Six tag SNVs of the *VDR* gene (rs7975232, rs1544410, rs2228570, rs731236, rs4516035, and rs11568820) were genotyped using the Multiplex SNaPshot system (Applied Biosystems, Foster City, USA). The forward (F), reverse (R), and extension primers used for the polymerase chain reaction (PCR) and extension reactions of the six SNVs are listed in Table 1. Detailed procedures of the SNaPshot genotyping method have been described previously [15].

### 2.3. Outcome Parameters

Outcome parameters included comparisons regarding mutant allele frequency, homozygous and heterozygous mutant versus homozygous wild, and the dominant and recessive models of the six *VDR* SNVs for the patients and healthy controls. In addition, clinical features of the FRI cohort were analysed. Furthermore, preoperative serum levels of white blood cell (WBC) count, percentage of polymorphonuclear leukocytes (PMN%), erythrocyte sedimentation rate (ESR), C-reactive protein (CRP), procalcitonin (PCT), interleukin-6 (IL-6), tumor necrosis factor-*α* (TNF-*α*), serum amyloid A (SAA), and total vitamin D among different genotypes of the *VDR* gene variation(s) with clinical significance in the patient group were compared.

### 2.4. Statistical Analysis

Statistical analysis was conducted using the Statistical Product and Service Solutions software (version 13.0, SPSS Inc., Chicago, IL, USA). Distributions of the continuous variables were first assessed for normality using the Kolmogorov-Smirnov test. Data were presented as mean ± standard deviation (SD) or median with interquartile range (IQR) based on data distribution. For normally distributed data, Student's *t*-test or one-way analysis of variance (ANOVA) was used to compare differences between two groups or among three groups. Otherwise, the Mann-Whitney or Kruskal-Wallis tests were applied. When using ANOVA, a test of homogeneity of variances was also conducted. When the assumption of the homogeneity of variances was met, the LSD method was used for post hoc multiple comparisons. Otherwise, the Welch's ANOVA and Dunnett's T3 method were applied for the whole and post hoc multiple comparisons, respectively, with statistical significance set at an adjusted *P* value less than 0.017 (three different genotype groups).

The genotype distributions of the healthy controls were tested to confirm the Hardy-Weinberg equilibrium (HWE) using the chi-square test. The chi-square test or Fisher's exact test was used to compare the genotype distributions and frequencies of mutant alleles and the four genetic models, with corresponding odds ratios (ORs) and 95% confidence intervals (CIs) between the patients and healthy controls. All reported values were 2-sided with a *P* value of less than 0.05, which was considered statistically significant.

## 3. Results

### 3.1. Demographics and FRI Characteristics

A total of 468 COM patients (357 males and 111 females) and 368 healthy controls (268 males and 100 females) were included, with no statistical differences regarding sex ratio (3.2 vs. 2.7, *P* = 0.25) and median age [48, IQR (33, 59) years vs. 46, IQR (37, 52) years, *P* = 0.08] between the two groups. Among the 468 patients, 70 and 62 patients were categorised as having diabetic foot-related and haematogenous spread-related infection, respectively, with the remaining 336 patients identified as having FRI. The mean age of the FRI patients at diagnosis was 43.3 ± 15.6 years. The clinical characteristics of the included FRI patients are depicted in Table 2.

### 3.2. HWE Test Outcomes of the Healthy Controls

All the genotyped *VDR* genetic variations were in the HWE among the healthy controls (*P* = 0.55 for rs7975232; *P* = 0.77 for rs1544410; *P* = 0.08 for rs2228570; *P* = 0.21 for rs731236; *P* = 0.63 for rs4516035; and *P* = 0.18 for rs11568820), demonstrating that the participants of the control group were representative.

### 3.3. Associations between *VDR* Genetic SNVs and the Risks of Developing FRI

Although no statistical difference was observed in the genotype distribution of rs7975232 between patients and healthy controls (*P* = 0.059), significant links were found between this SNV site and risk of FRI development in the recessive model (OR = 0.530, 95% CI 0.310–0.906, *P* = 0.019) and homozygous models (OR = 0.515, 95% CI 0.295–0.898, *P* = 0.018), suggesting that people with the AA genotype at this site were less susceptible to FRI (Table 3).

With respect to the other five SNV sites, although no statistical differences were detected, individuals with genotype CT of rs1544410 and GG and AG of rs2228570 might be at a lower risk, whereas people with the CT genotype of rs4516035 might be at a higher risk of developing FRI (Table 3).

### 3.4. Stratified Analyses Regarding Links between *VDR* Genetic SNVs and the Risks of FRI Development by Sex and Age

As shown in Table S1, rs7975232 may also be associated to decreased susceptibility to FRI in males and patients below 60 years, demonstrating that such groups of people with the AA genotype were in a lower risk to develop FRI. In addition to rs7975232, rs2228570 may be also related to reduced risk of FRI development in females and patients below 60 years, indicating that such groups of people with the GG/AG genotypes were less susceptible to FRI. However, no significant relationships were found between the remaining *VDR* genetic SNVs and the risks of developing FRI in this cohort.

### 3.5. Preoperative Serum Levels of Inflammatory Biomarkers and Total Vitamin D among Different Genotypes of the Six *VDR* SNV Sites among the FRI Patients

Significant differences were found in the preoperative serological level of total vitamin D (*P* = 0.021) among different genotypes of rs7975232. Outcomes of post hoc multiple comparisons revealed that FRI patients with genotype AA had a relatively higher level of total vitamin D than those with AC and CC genotypes. Although no statistical differences were observed in serum TNF-*α* levels among the three groups (*P* = 0.068), patients with AA genotype had relatively lower TNF-*α* levels than those with AC (*P* = 0.022) and CC genotypes (*P* = 0.031). No significant differences were identified in the mean levels of WBC, PMN, ESR, CRP, PCT, IL-6, or SAA among the three genotypes of rs7975232 in patients with FRI (Table 4). Comparison outcomes regarding serum levels of the 8 inflammatory biomarkers and total vitamin D among different genotypes of another five SNV sites were listed in the supplemental table (Table S2).

## 4. Discussion

The results of the current case-control study, comprising 704 Chinese participants, demonstrated that the *VDR* gene sequence variant *ApaI* (rs7975232) was associated with a decreased risk of late FRI development, and people with the AA genotype were less susceptible to FRI. In addition, we found that patients with AA genotype had a higher total vitamin D level than those with the AC and CC genotypes, implying vitamin D may have a protective effect against bone infection. Aside from *ApaI*, we also observed that *BsmI*, *FokI*, and *GATA* variations of *VDR* may also be correlated with the risks of developing FRI, which needs to be confirmed by further studies.

The present study shared similarities and differences with a previous study [15], which also investigated *VDR* gene variations and susceptibility to bone infection. First, this study focused on late FRI only, whereas the previous study recruited different types of COM patients, which also included those with infections following haematogenous spread and diabetic foot. Second, the sample size of this study was larger than the previous one. Third, in addition to the previously analysed six inflammatory biomarkers, ESR, PMN%, and vitamin D levels were also compared among different genotypes, which provided a direct insight into the effects of *VDR* SNV on inflammatory cytokines and vitamin D levels. Our findings are discussed using the following three aspects.

First, we found that *ApaI* (rs7975232) may be associated with a decreased risk of FRI development in this cohort, with AA genotype as a protective factor. In the stratified analyses by sex and age, we found that this SNV was linked to FRI development in males and patients under 60 years. In a previous study, we did not find any significant correlation between *ApaI* and COM, which might be related to the limited sample size and the inclusion of different types of COM in the study [15]. To evaluate the potential effects of such genetic variation, preoperative levels of eight inflammatory biomarkers or cytokines and total vitamin D were compared among the different *ApaI* genotypes. The results showed that patients with AA genotype had a relatively higher vitamin D level than those of the AC and CC genotypes, implying that an elevated vitamin D level may play important roles against FRI. One of the underlying mechanisms that resulted in the AA genotype of rs7975232 having a relatively higher vitamin D level may due to the fact that this SNV site is located at the 3′-end of the *VDR* gene, which associates with different lengths of the polyadenylate sequence and affects the mRNA stability and, therefore, different vitamin D levels [19]. In addition, although no statistical differences were observed, FRI patients with AA genotype also had relatively lower levels of WBC, ESR, PCT, TNF-*α*, and SAA than those with the AC and CC genotypes. Similar to our results, a recent meta-analysis found that rs7975232 was related to a reduced risk of pulmonary tuberculosis (PTB) in the African population [20]. In addition to bacterial infection, a previous study also found that rs7975232 was linked to a decreased susceptibility to hepatitis C virus (HCV) infection in a Chinese population, and the authors also indicated that low vitamin D levels may increase the risk of HCV infection and chronicity [21], which is in accordance with the present study.

Second, we found that *BsmI* (rs1544410) and *FokI* (rs2228570) might also be correlated with decreased risk of FRI development, although no statistical differences were observed. In the subgroup analyses by sex and age, we also noted that *FokI* was linked to FRI development in females and patients under 60 years. Several studies have also found positive links between the two sites and susceptibility to infectious diseases. Based on a meta-analysis of 19 studies comprising 3,644 controls and 2,635 patients, Areeshi et al. found that rs1544410 may be a risk factor for PTB in the Asian population [22], which was supported by a subsequent meta-analysis focusing on the Iranian population [23]. In addition to PTB, Shaker et al. also found that rs1544410 could be regarded as a predictor of response to combination therapy with HCV [24]. With respect to rs2228570, a recent case-control study revealed that *FokI* increased the risk of community-acquired pneumonia (CAP) development in Indian children [25]. Additionally, this SNV site was also found to be related to an elevated risk of neonatal sepsis, with the TT genotype having a relatively lower 25-hydroxyvitamin D (25OHD) level, implying that this genetic variation may participate in sepsis via its influence on peripheral vitamin D levels. Although no statistical results were obtained in our study, there was a tendency for both SNVs to be associated with decreased risks of FRI development, with the CT genotype of rs1544410 and the GG and AG genotypes of rs2228570 as underlying protective factors. Therefore, to obtain more accurate conclusions, future studies with larger sample sizes are necessary.

Third, we found that *GATA* (rs4516035) might increase susceptibility to FRI, although no statistical difference was obtained, with the CT genotype as a possible risk factor. In contrast to the previous four *VDR* genetic variations, the number of studies focusing on the potential relationship between this SNV site and the development of infectious disorders remains limited. Nonetheless, this site was found to be positively linked to muscle strength [26] and type 1 diabetes in children [27], the latter of which was achieved via its influence on serum 25OHD concentration during pregnancy in mothers. Regarding *Cdx-2* (rs11568820), we did not find any positive association between this site and FRI development, and it should be noted that the results of SNV studies are influenced by several factors apart from the sample size, such as ethnicity, selection of controls, diagnostic criteria, and detection method. Therefore, care should be taken when looking at the outcomes from a single report, and definite conclusions should be drawn on studies with a high evidence level, such as high-quality meta-analyses and systematic reviews.

Although in the past few decades, several investigations have reported relationships between *VDR* genetic variations and the risk of developing inflammatory disorders, limitations still exist. First, the sample size of most studies was limited, which directly affected the reliability of the outcomes. Second, the number of studies reporting the effects of *VDR* SNVs on serum vitamin D levels remains limited. Although our study revealed that patients with genotype AA of rs7975232 had a relatively higher vitamin D level than AC and CC genotypes, the median levels of serological vitamin D of all the three groups showed insufficient. In addition, as vitamin D levels of the healthy controls were not detected here and, also, FRI is a multifactorial-related disorder, definite conclusion cannot be made that FRI development is related or not related to vitamin D level based on the present results. Third, the detailed mechanisms of *VDR* genetic alterations in the pathogenesis of inflammation or infection remain largely unknown.

The present study had some limitations. First, although the sample size of this study was larger than that of most previous studies on FRI, it was still limited for an SNV investigation. Thus, more participants should be included in order to achieve more reliable outcomes, particularly for the *BsmI*, *FokI*, and *GATA* SNV sites. Second, although we analysed preoperative levels of the inflammatory biomarkers and vitamin D, potential influences of confounding factors such as previous interventions, seasonal influence, and diet cannot be neglected. Although we explored the potential effects of *ApaI* SNV on serological levels of different biomarkers, it is still far from sufficient, and further investigation should also focus more on serological indicators apart from vitamin D, such as calcium, phosphate, and alkaline phosphatase levels. Also, in-depth research is required to uncover potential mechanisms in detail. Third, as FRI is a highly heterogeneous disorder, the occurrence of which is associated with complex interactions between external and internal factors, our study only analysed its pathogenesis from the perspective of genetic predisposition, which created a bias. Another important factors, such as cigarette smoking and alcohol consumption, cannot be ignored, either. Nonetheless, it is still valuable as it certifies that genetic predisposition is also involved in the development of late FRI.

## 5. Conclusions

In summary, in this Chinese cohort, *ApaI* was found to be associated with a decreased risk of developing late FRI, with the AA genotype as a protective factor. Patients with the AA genotype at *ApaI* had a relatively higher level of vitamin D than AC and CC genotypes. Although there were tendencies regarding *BsmI*, *FokI*, and *GATA* variations and FRI development, there is still a lack of statistical support. The underlying mechanisms of *VDR* SNVs in FRI pathogenesis should be further explored.

## Figures and Tables

**Table 1:** PCR primers and extension primers of the six *VDR* genetic variations.

SNVs	PCR primers	Extension primers
rs7975232	F: 5′-GGCACGGGGATAGAGAAGAA-3′	5′-CTGACTGACTGACTGACTCACAGGAGCTCTCAGCTGGGC-3′
	R: 5′-GCACGGAGAAGTCACTGGA-3′	
rs1544410	F: 5′-GTGCAGGCGATTCGTAGG-3′	5′-TGGGGCCACAGACAGGCCTGC-3′
	R: 5′-ACCATCTCTCAGGCTCCAAA-3′	
rs2228570	F: 5′-GCACTGACTCTGGCTCTGA-3′	5′-CTGACTGACTGACTGACTGACTGACTCTTGCTGTTCTTACAGGGA-3′
	R: 5′-GCCTTCACAGGTCATAGCATTG-3′	
rs731236	F: 5′-TGGTGGGATTGAGCAGTGA-3′	5′-GCAGGACGCCGCGCTGAT-3′
	R: 5′-GAAGGAGAGGCAGCGGTA-3′	
rs4516035	F: 5′-CCTCTTCTTAGAACTCACTGTGC-3′	5′-TCCTTTAGCCAGGGAAGA-3′
	R: 5′-CCTTGTCCCTCTGAGCCAT-3′	
rs11568820	F: 5′-AGAACATCTTTTGTATCAGGAACT-3′	5′-CTGACTGACTGACTGACTGACTGACTCCTGAGTAAACTAGGTCACA-3′
	R: 5′-AATGTAAGAAGCTGTAGCAATGAA-3′	

**Table 2:** Clinical characteristics of the FRI patients included.

Clinical characteristics	Outcomes
Top injury type (no., %)	
Traffic accident	93 (40.0%)
Falling injury	50 (21.5%)
Falling from a height	27 (11.6%)
Stab injury	21 (9.0%)
Body side distribution (no., %)	
Left	159 (47.3%)
Right	170 (50.6%)
Bilateral	7 (2.1%)
Single infection site (no., %)	268 (79.8%)
Tibia	158 (59.0%)
Femur	41 (15.3%)
Calcaneus	28 (10.4%)
Foot	8 (3.0%)
Hand	7 (2.6%)
Fibula	7 (2.6%)
Humerus	6 (2.2%)
Ulna	5 (1.9%)
Radius	4 (1.5%)
Another sites	4 (1.5%)
Positive rate of intraoperative culture (%, no.)	57.1% (132/231)
Monomicrobial infection (%, no.)	63.6% (84/132)
Top detected pathogens for monomicrobial infection (no., %)	
*Staphylococcus aureus*	39 (46.4%)
*Pseudomonas aeruginosa*	12 (14.3%)
*Staphylococcus epidermidis*	7 (8.3%)
*Enterobacter cloacae*	6 (7.1%)
*Enterococcus faecalis*	5 (6.0%)

**Table 3:** Associations between the six *VDR* genetic SNVs and susceptibilities to late FRI.

SNVs	Allele or genotype	Patients (*n* = 336)	Controls (*n* = 368)	*P* values	OR (95% CI)
rs7975232	C	480 (71.4%)	492 (66.8%)		Ref.
	A	192 (28.6%)	244 (33.2%)	0.063	0.807 (0.643-1.012)
	CC	166 (49.4%)	167 (45.4%)		Ref.
	AC	148 (44.0%)	158 (42.9%)	0.708	0.942 (0.691-1.285)
	AA	22 (6.6%)	43 (11.7%)	0.018	0.515 (0.295-0.898)
	Dominant (AA+AC vs. CC)			0.285	0.851 (0.633-1.144)
	Recessive (AA vs. AC+CC)			0.019	0.530 (0.310-0.906)
rs1544410	C	645 (96.0%)	692 (94.0%)		Ref.
	T	27 (4.0%)	44 (6.0%)	0.093	0.658 (0.403-1.076)
	CC	310 (92.3%)	325 (88.3%)		Ref.
	CT	25 (7.4%)	42 (11.4%)	0.073	0.624 (0.371-1.049)
	TT	1 (0.3%)	1 (0.3%)	1.000	1.048 (0.065-16.834)
	Dominant (TT+CT vs. CC)			0.079	0.634 (0.380-1.057)
	Recessive (TT vs. CT+CC)			1.000	1.096 (0.068-17.584)
rs2228570	A	333 (49.6%)	331 (45.0%)		Ref.
	G	339 (50.4%)	405 (55.0%)	0.085	0.832 (0.675-1.026)
	AA	80 (23.8%)	66 (17.9%)		Ref.
	AG	173 (51.5%)	199 (54.1%)	0.090	0.717 (0.488-1.053)
	GG	83 (24.7%)	103 (28.0%)	0.066	0.665 (0.430-1.028)
	Dominant (GG+AG vs. AA)			0.055	0.699 (0.485-1.008)
	Recessive (GG vs. AG+AA)			0.323	0.844 (0.603-1.182)
rs731236	A	647 (96.3%)	700 (95.1%)		Ref.
	G	25 (3.7%)	36 (4.9%)	0.281	0.751 (0.446-1.265)
	AA	311 (92.6%)	334 (90.8%)		Ref.
	AG	25 (7.4%)	32 (8.7%)	0.528	0.839 (0.486-1.448)
	GG	0 (0.0%)	2 (0.5%)	0.500	1.006 (0.998-1.014)
	Dominant (GG+AG vs. AA)			0.390	0.790 (0.461-1.354)
	Recessive (GG vs. AG+AA)			0.500	1.005 (0.998-1.013)
rs4516035	T	644 (95.8%)	718 (97.6%)		Ref.
	C	28 (4.2%)	18 (2.4%)	0.070	1.734 (0.950-3.165)
	TT	308 (91.7%)	350 (95.1%)		Ref.
	CT	28 (8.3%)	18 (4.9%)	0.065	1.390 (0.772-2.504)
	CC	0 (0.0%)	0 (0.0%)	N/A	N/A
	Dominant (CC+CT vs. TT)			0.065	1.768 (0.959-3.259)
	Recessive (CC vs. CT+TT)			N/A	N/A
rs11568820	C	380 (56.5%)	432 (58.7%)		Ref.
	T	292 (43.5%)	304 (41.3%)	0.415	1.092 (0.884-1.349)
	CC	106 (31.5%)	133 (36.1%)		Ref.
	CT	168 (50.0%)	166 (45.1%)	0.160	1.270 (0.910-1.772)
	TT	62 (18.5%)	69 (18.8%)	0.582	1.127 (0.735-1.729)
	Dominant (TT+CT vs. CC)			0.199	1.228 (0.898-1.680)
	Recessive (TT vs. CT+CC)			0.919	0.981 (0.670-1.434)

FRI: fracture-related infection; SNV: single nucleotide variation; OR: odds ratio, CI: confidence interval; N/A: not available.

**Table 4:** Preoperative serological levels of inflammatory biomarkers and vitamin D among different genotypes of rs7975232 in FRI patients.

rs7975232	WBC (×10^9^/l)	PMN (%)	ESR (mm/1 h)	CRP (mg/l)	PCT (ng/ml)	IL-6 (pg/ml)	TNF-*α* (pg/ml)	SAA (mg/l)	Vitamin D (ng/ml)
AA	6.0 (5.2, 7.6)	59.5 (53.9, 63.3)	14 (7.5, 48.5)	4.6 (1.8, 14.2)	0.037 (0.029, 0.082)	7.3 (3.7, 13.8)	8.1 (6.8, 9.3)	8.5 (5.0, 28.2)	20.6 (19.6, 24.6)
AC	7.1 (5.6, 8.3)	59.2 (50.1, 65.5)	16 (8, 33)	4.5 (1.5, 10.4)	0.045 (0.034, 0.067)	5.9 (3.5, 10.7)	9.3 (7.6, 11.8)	10.7 (6.4, 31.4)	20.3 (15.0, 24.0)
CC	6.9 (5.8, 8.4)	60.0 (51.8, 66.9)	15 (7.0, 36.8)	3.9 (1.4, 13.1)	0.043 (0.027, 0.068)	6.1 (3.4, 12.7)	9.4 (7.6, 11.9)	10.8 (6.2, 19.1)	17.9 (13.5, 22.9)
*P* values^∗^	0.257	0.779	0.639	0.933	0.745	0.833	0.068	0.632	0.021
Post hoc multiple comparisons^#^									
AA vs. AC	0.110	0.981	0.863	0.715	0.897	0.563	0.022	0.330	0.286
AA vs. CC	0.113	0.698	0.561	0.737	0.654	0.557	0.031	0.403	0.019
AC vs. CC	0.923	0.505	0.390	0.925	0.471	0.963	0.969	0.826	0.035

FRI: fracture-related infection; WBC: white blood cell count; PMN%: percentage of polymorphonuclear; ESR: erythrocyte sedimentation rate; CRP: C-reactive protein; PCT: procalcitonin; IL-6: interleukin-6; TNF-*α*: tumor necrosis factor-*α*; SAA: serum amyloid A. ^∗^These *P* values were obtained by the Kruskal-Wallis H test. ^#^The alpha level was 0.017 for the post hoc multiple comparisons.

## Data Availability

The datasets generated and/or analysed during the current study are not publicly available due to the respect and protection of privacy of the patients but are available from the corresponding authors on reasonable request.

## Supplementary Materials

Supplementary 1 Table S1: stratified analyses regarding associations between the six *VDR* genetic SNVs and susceptibilities to late FRI by sex and age.

Supplementary 2 Table S2: preoperative serological levels of inflammatory biomarkers and vitamin D among different genotypes of rs1544410, rs2228570, rs731236, rs4516035, and rs11568820 in the FRI patients included.
