# Supplementary material for: Vitamin D Receptor Genetic Variations May Associate with the Risk of Developing Late Fracture-Related Infection in the Chinese Han Population
Source: J Immunol Res. 2022 Feb 10;2022:9025354. doi: 10.1155/2022/9025354 (PMC8886694; doi:10.1155/2022/9025354)
Supplement: Supplementary 1 — Table S1: stratified analyses regarding associations between the six VDR genetic SNVs and susceptibilities to late FRI by sex and age. [file 9025354.f1.docx]

**Table S1**

**Stratified analyses regarding associations between the six *VDR* genetic SNVs and susceptibilities to late FRI by sex and age**

| SNVs | Allele or Genotype | Patients | Controls | *P* values | OR (95% CI) |
| --- | --- | --- | --- | --- | --- |
| **Males** |  | **N = 273** | **N = 268** |  |  |
| rs7975232 | CC | 137 (50.2%) | 120 (44.8%) |  | Ref. |
|  | AC | 118 (43.2%) | 118 (44.0%) | 0.463 | 0.876 (0.615 - 1.248) |
|  | AA | 18 (6.6%) | 30 (11.2%) | **0.044** | 0.526 (0.279 - 0.990) |
|  | Dominant (AA+AC vs. CC) | | | 0.208 | 0.805 (0.574 - 1.129) |
|  | Recessive (AA vs. AC+CC) | | | **0.060** | 0.560 (0.304 - 1.031) |
| rs1544410 | CC | 253 (92.7%) | 244 (91.0%) |  | Ref. |
|  | CT | 19 (6.9%) | 24 (9.0%) | 0.398 | 0.764 (0.408 -1.429) |
|  | TT | 1 (0.4%) | 0 (0.0%) | N/A | N/A |
|  | Dominant (TT+CT vs. CC) | | | 0.488 | 0.804 (0.433 - 1.492) |
|  | Recessive (TT vs. CT+CC) | | | N/A | N/A |
| rs2228570 | AA | 64 (23.4%) | 50 (18.7%) |  | Ref. |
|  | AG | 139 (50.9%) | 148 (55.2%) | 0.164 | 0.734 (0.474 - 1.135) |
|  | GG | 70 (25.7%) | 70 (26.1%) | 0.330 | 0.781 (0.475 - 1.284) |
|  | Dominant (GG+AG vs. AA) | | | 0.172 | 0.749 (0.494 - 1.135) |
|  | Recessive (GG vs. AG+AA) | | | 0.899 | 0.975 (0.664 - 1.433) |
| rs731236 | AA | 253(92.7%) | 250 (93.3%) |  | Ref. |
|  | AG | 20 (7.3%) | 18 (6.7%) | 0.781 | 1.098 (0.567 - 2.125) |
|  | GG | 0 (0.0%) | 0 (0.0%) | N/A | N/A |
|  | Dominant (GG+AG vs. AA) | | | 0.781 | 1.098 (0.567 - 2.125) |
|  | Recessive (GG vs. AG+AA) | | | N/A | N/A |
| rs4516035 | TT | 250 (91.6%) | 254 (94.8%) |  | Ref. |
|  | CT | 23 (8.4%) | 14 (5.2%) | 0.140 | 1.669 (0.840 - 3.318) |
|  | CC | 0 (0.0%) | 0 (0.0%) | N/A | N/A |
|  | Dominant (CC+CT vs. TT) | | | 0.140 | 1.669 (0.840 - 3.318) |
|  | Recessive (CC vs. CT+TT) | | | N/A | N/A |
| rs11568820 | CC | 86 (31.5%) | 101 (37.7%) |  | Ref. |
|  | CT | 131 (48.0%) | 112 (41.8%) | 0.103 | 1.374 (0.937 - 2.014) |
|  | TT | 56 (20.5%) | 55 (20.5%) | 0.456 | 1.196 (0.747 - 1.914) |
|  | Dominant (TT+CT vs. CC) | | | 0.132 | 1.314 (0.921 - 1.878) |
|  | Recessive (TT vs. CT+CC) | | | 0.998 | 0.999 (0.658 - 1.517) |
| **Females** |  | **N = 63** | **N = 100** |  |  |
| rs7975232 | CC | 29 (46.0%) | 47 (47.0%) |  | Ref. |
|  | AC | 30 (47.6%) | 40 (40.0%) | 0.563 | 1.216 (0.627 - 2.357) |
|  | AA | 4 (6.4%) | 13 (13.0%) | 0.254 | 0.499 (0.148 - 1.676) |
|  | Dominant (AA+AC vs. CC) | | | 0.904 | 1.040 (0.553 - 1.956) |
|  | Recessive (AA vs. AC+CC) | | | 0.176 | 0.454 (0.141 - 1.460) |
| rs1544410 | CC | 57 (90.5%) | 81 (81.0%) |  | Ref. |
|  | CT | 6 (9.5%) | 18 (18.0%) | 0.130 | 0.474 (0.177 - 1.267) |
|  | TT | 0 (0.0%) | 1 (1.0%) | N/A | N/A |
|  | Dominant (TT+CT vs. CC) | | | 0.102 | 0.449 (0.169 - 1.194) |
|  | Recessive (TT vs. CT+CC) | | | N/A | N/A |
| rs2228570 | AA | 16 (25.4%) | 16 (16.0%) |  | Ref. |
|  | AG | 34 (54.0%) | 51 (51.0%) | 0.330 | 0.667 (0.294 - 1.510) |
|  | GG | 13 (20.6%) | 33 (33.0%) | **0.051** | 0.394 (0.153 - 1.013) |
|  | Dominant (GG+AG vs. AA) | | | 0.141 | 0.560 (0.257 - 1.220) |
|  | Recessive (GG vs. AG+AA) | | | **0.088** | 0.528 (0.252 - 1.105) |
| rs731236 | AA | 58 (92.1%) | 84 (84.0%) |  | Ref. |
|  | AG | 5 (7.9%) | 14 (14.0%) | 0.223 | 0.517 (0.177 - 1.515) |
|  | GG | 0 (0.0%) | 2 (2.0%) | N/A | N/A |
|  | Dominant (GG+AG vs. AA) | | | 0.135 | 0.453 (0.157 - 1.304) |
|  | Recessive (GG vs. AG+AA) | | | N/A | N/A |
| rs4516035 | TT | 58 (92.1%) | 96 (96.0%) |  | Ref. |
|  | CT | 5 (7.9%) | 4 (4.0%) | 0.309 | 2.069 (0.534 - 8.018) |
|  | CC | 0 (0.0%) | 0 (0.0%) | N/A | N/A |
|  | Dominant (CC+CT vs. TT) | | | 0.309 | 2.069 (0.534 - 8.018) |
|  | Recessive (CC vs. CT+TT) | | | N/A | N/A |
| rs11568820 | CC | 20 (31.7%) | 32 (32.0%) |  | Ref. |
|  | CT | 37 (58.7%) | 54 (54.0%) | 0.796 | 1.096 (0.546 - 2.203) |
|  | TT | 6 (9.5%) | 14 (14.0%) | 0.503 | 0.686 (0.227 - 2.076) |
|  | Dominant (TT+CT vs. CC) | | | 0.973 | 1.012 (0.514 - 1.991) |
|  | Recessive (TT vs. CT+CC) | | | 0.396 | 0.647 (0.235 - 1.781) |
| **Age ≥ 60 years** | | **N = 55** | **N = 13** |  |  |
| rs7975232 | CC | 26 (47.3%) | 5 (38.5%) |  | Ref. |
|  | AC | 27 (49.1%) | 6 (46.2%) | 0.828 | 0.865 (0.235 - 3.186) |
|  | AA | 2 (3.6%) | 2 (15.4%) | 0.171 | 0.192 (0.022 - 1.703) |
|  | Dominant (AA+AC vs. CC) | | | 0.566 | 0.697 (0.202 - 2.400) |
|  | Recessive (AA vs. AC+CC) | | | 0.162 | 0.208 (0.026 - 1.636) |
| rs1544410 | CC | 50 (90.9%) | 11 (84.6%) |  | Ref. |
|  | CT | 5 (9.1%) | 2 (15.4%) | 0.611 | 0.550 (0.094 – 3.213) |
|  | TT | 0 (0.0%) | 0 (0.0%) | N/A | N/A |
|  | Dominant (TT+CT vs. CC) | | | 0.611 | 0.550 (0.094 – 3.213) |
|  | Recessive (TT vs. CT+CC) | | | N/A | N/A |
| rs2228570 | AA | 10 (18.2%) | 4 (30.8%) |  | Ref. |
|  | AG | 29 (52.7%) | 8 (61.5%) | 0.715 | 1.450 (0.358 - 5.874) |
|  | GG | 16 (29.1%) | 1 (7.7%) | 0.148 | 6.400 (0.623 - 65.739) |
|  | Dominant (GG+AG vs. AA) | | | 0.445 | 2.000 (0.512 - 7.813) |
|  | Recessive (GG vs. AG+AA) | | | 0.160 | 4.923 (0.590 - 41.071) |
| rs731236 | AA | 52 (94.5%) | 11 (84.6%) |  | Ref. |
|  | AG | 3 (5.5%) | 2 (15.4%) | 0.241 | 0.317 (0.047 - 2.129) |
|  | GG | 0 (0.0%) | 0 (0.0%) | N/A | N/A |
|  | Dominant (GG+AG vs. AA) | | | 0.241 | 0.317 (0.047 - 2.129) |
|  | Recessive (GG vs. AG+AA) | | | N/A | N/A |
| rs4516035 | TT | 48 (87.3%) | 13 (100.0%) |  | Ref. |
|  | CT | 7 (12.7%) | 0 (0.0%) | N/A | N/A |
|  | CC | 0 (0.0%) | 0 (0.0%) | N/A | N/A |
|  | Dominant (CC+CT vs. TT) | | | N/A | N/A |
|  | Recessive (CC vs. CT+TT) | | | N/A | N/A |
| rs11568820 | CC | 21 (38.2%) | 0 (0.0%) |  | Ref. |
|  | CT | 23 (41.8%) | 0 (0.0%) | N/A | N/A |
|  | TT | 11 (20.0%) | 13 (100.0%) | N/A | N/A |
|  | Dominant (TT+CT vs. CC) | | | N/A | N/A |
|  | Recessive (TT vs. CT+CC) | | | N/A | N/A |
| **Age ＜ 60 years** | | **N = 281** | **N = 355** |  |  |
| rs7975232 | CC | 140 (49.8%) | 162 (45.6%) |  | Ref. |
|  | AC | 121 (43.1%) | 152 (42.8%) | 0.624 | 0.921 (0.663 - 1.280) |
|  | AA | 20 (7.1%) | 41 (11.5%) | **0.052** | 0.564 (0.316 - 1.009) |
|  | Dominant (AA+AC vs. CC) | | | 0.294 | 0.845 (0.618 - 1.157) |
|  | Recessive (AA vs. AC+CC) | | | **0.059** | 0.587 (0.335 - 1.027) |
| rs1544410 | CC | 260 (92.5%) | 314 (88.5%) |  | Ref. |
|  | CT | 20 (7.1%) | 40 (11.3%) | 0.076 | 0.604 (0.344 - 1.059) |
|  | TT | 1 (0.4%) | 1 (0.3%) | 1.000 | 1.208 (0.075 - 19.402) |
|  | Dominant (TT+CT vs. CC) | | | 0.085 | 0.619 (0.357 - 1.073) |
|  | Recessive (TT vs. CT+CC) | | | 1.000 | 1.264 (0.079 - 20.302) |
| rs2228570 | AA | 70 (24.9%) | 62 (17.5%) |  | Ref. |
|  | AG | 144 (51.2%) | 191 (53.8%) | **0.050** | 0.668 (0.446 - 1.001) |
|  | GG | 67 (23.8%) | 102 (28.7%) | **0.021** | 0.582 (0.367 - 0.922) |
|  | Dominant (GG+AG vs. AA) | | | **0.022** | 0.638 (0.434 - 0.937) |
|  | Recessive (GG vs. AG+AA) | | | 0.166 | 0.777 (0.543 - 1.111) |
| rs731236 | AA | 259 (92.2%) | 323 (91.0%) |  | Ref. |
|  | AG | 22 (7.8%) | 30 (8.5%) | 0.760 | 0.915 (0.515 - 1.624) |
|  | GG | 0 (0.0%) | 2 (0.6%) | N/A | N/A |
|  | Dominant (GG+AG vs. AA) | | | 0.594 | 0.857 (0.486 - 1.511) |
|  | Recessive (GG vs. AG+AA) | | | N/A | N/A |
| rs4516035 | TT | 260 (92.5%) | 337 (94.9%) |  | Ref. |
|  | CT | 21 (7.5%) | 18 (5.1%) | 0.210 | 1.512 (0.789 - 2.897) |
|  | CC | 0 (0.0%) | 0 (0.0%) | N/A | N/A |
|  | Dominant (CC+CT vs. TT) | | | 0.210 | 1.512 (0.789 - 2.897) |
|  | Recessive (CC vs. CT+TT) | | | N/A | N/A |
| rs11568820 | CC | 85 (30.2%) | 125 (35.2%) |  | Ref. |
|  | CT | 145 (51.6%) | 162 (45.6%) | 0.129 | 1.316 (0.923 - 1.878) |
|  | TT | 51 (18.1%) | 68 (19.2%) | 0.673 | 1.103 (0.699 - 1.740) |
|  | Dominant (TT+CT vs. CC) | | | 0.186 | 1.253 (0.896 - 1.752) |
|  | Recessive (TT vs. CT+CC) | | | 0.747 | 0.936 (0.626 - 1.399) |

FRI: Fracture related infection; SNV: single nucleotide variation; OR: odds ratio, CI: confidence interval; N/A: not available.
