# Supplementary material for: Vitamin D Receptor Genetic Variations May Associate with the Risk of Developing Late Fracture-Related Infection in the Chinese Han Population
Source: J Immunol Res. 2022 Feb 10;2022:9025354. doi: 10.1155/2022/9025354 (PMC8886694; doi:10.1155/2022/9025354)
Supplement: Supplementary 2 — Table S2: preoperative serological levels of inflammatory biomarkers and vitamin D among different genotypes of rs1544410, rs2228570, rs731236, rs4516035, and rs11568820 in the FRI patients included. [file 9025354.f2.docx]

**Table S2**

**Preoperative serological levels of inflammatory biomarkers and vitamin D among different genotypes of rs1544410, rs2228570, rs731236, rs4516035 and rs11568820 in the FRI patients included**

| Items | WBC (×10^9^/L) | PMN% (%) | ESR (mm/1h) | CRP (mg/L) | PCT (ng/ml) | IL-6 (pg/ml) | TNF-α (pg/ml) | SAA (mg/L) | Vitamin D (ng/mL) |
| --- | --- | --- | --- | --- | --- | --- | --- | --- | --- |
| **rs1544410** |  |  |  |  |  |  |  |  |  |
| CC | 7.0 (5.7, 8.3) | 59.7 (51.9, 66.8) | 15 (7, 40) | 4.3 (1.4, 12.4) | 0.045 (0.032, 0.071) | 6.0 (3.5, 11.5) | 9.4 (7.6, 11.9) | 10.7 (6.2, 24.2) | 19.8 (14.2, 23.5) |
| CT | 6.6 (5.5, 8.0) | 54.9 (50.0, 63.2) | 15 (7.8, 24.5) | 4.9 (2.0, 8.8) | 0.030 (0.028, 0.038) | 6.9 (3.0, 14.0) | 8.3 (7.2, 9.7) | 11.8 (8.2, 30.4) | 18.8 (14.7, 27.6) |
| TT | 4.5 | 49.3 | 4 | 2.3 | 0.030 | / | 7.73 | 3.8 | 22.0 |
| *P* values * | 0.264 | 0.101 | 0.698 | 0.923 | 0.017 | 0.897 | 0.081 | 0.573 | 0.631 |
| **rs2228570** |  |  |  |  |  |  |  |  |  |
| AA | 7.4 (5.8, 9.1) | 61.0 (54.0, 68.2) | 16 (7, 41) | 3.6 (1.3, 11.5) | 0.044 (0.034, 0.073) | 6.0 (3.8, 12.1) | 9.9 (7.6, 12.2) | 10.3 (6.2, 35.5) | 17.9 (13.3, 22.5) |
| AG | 6.9 (5.6, 8.2) | 59.9 (51.5, 66.6) | 14 (6, 35.3) | 4.6 (1.5, 10.5) | 0.042 (0.031, 0.064) | 6.5 (3.3, 13.8) | 9.1 (7.5, 11.3) | 10.7 (6.3, 27.2) | 20.5 (15.6, 24.2) |
| GG | 6.7 (5.7, 8.2) | 57.2 (50.8, 65.3) | 16 (10, 42) | 4.4 (1.8, 12.8) | 0.048 (0.028, 0.079) | 5.6 (3.4, 9.9) | 9.3 (7.6, 12.0) | 11.1 (6.1, 23.4) | 18.8 (13.3, 21.9) |
| *P* values ^#^ | 0.295 | 0.217 | 0.299 | 0.755 | 0.820 | 0.669 | 0.645 | 0.979 | 0.017 |
| Post hoc multiple comparisons ^¶^ | | | | | | | | | |
| AA vs. AG | 0.134 | 0.467 | 0.490 | 0.507 | 0.510 | 0.696 | 0.373 | 0.866 | **0.014** |
| AA vs. GG | 0.223 | 0.087 | 0.529 | 0.532 | 0.923 | 0.360 | 0.647 | 0.943 | 0.794 |
| AG vs. GG | 0.878 | 0.200 | 0.116 | 0.809 | 0.785 | 0.540 | 0.621 | 0.870 | 0.030 |
| **rs731236** |  |  |  |  |  |  |  |  |  |
| AA | 7.0 (5.7, 8.3) | 59.8 (51.9, 66.8) | 16 (7, 40) | 4.3 (1.4, 12.4) | 0.045 (0.032, 0.071) | 6.0 (3.5, 12.1) | 9.4 (7.7, 11.9) | 10.8 (6.2, 24.3) | 19.9 (14.2, 23.5) |
| AG | 6.1 (5.4, 7.8) | 54.9 (50.2, 62.9) | 13 (5, 23.5) | 4.6 (1.7, 8.7) | 0.031 (0.028, 0.039) | 6.5 (3.2, 12.1) | 8.0 (7.2, 9.2) | 9.5 (7.9, 26.7) | 18.8 (14.6, 25.2) |
| *P* values ^^^ | 0.160 | 0.065 | 0.341 | 0.819 | 0.031 | 0.811 | 0.021 | 0.911 | 0.857 |
| **rs4516035** |  |  |  |  |  |  |  |  |  |
| CT | 6.7 (5.5, 8.7) | 60 (52.7, 66.8) | 25 (12, 55) | 4.6 (1.8, 19.2) | 0.040 (0.032, 0.050) | 7.6 (2.9, 19.0) | 9.3 (8.1, 12.4) | 9.7 (6.0, 42.8) | 17.8 (13.1, 21.4) |
| TT | 7.0 (5.7, 8.3) | 59.4 (51.6, 66.5) | 14 (7, 34.5) | 4.3 (1.5, 11.2) | 0.044 (0.030, 0.071) | 6.0 (3.5, 11.5) | 9.3 (7.5, 11.8) | 10.8 (6.2, 23.0) | 20.0 (14.4, 23.8) |
| *P* values ^^^ | 0.564 | 0.480 | 0.098 | 0.242 | 0.449 | 0.424 | 0.476 | 0.795 | 0.237 |
| **rs11568820** |  |  |  |  |  |  |  |  |  |
| CC | 6.8 (5.7, 8.0) | 59.6 (52.8, 64.2) | 12 (7, 22) | 3.2 (1.4, 8.9) | 0.041 (0.030, 0.070) | 4.8 (2.7, 9.0) | 8.8 (7.2, 11.0) | 9.7 (5.1, 18.9) | 20.4 (14.7, 23.7) |
| CT | 6.9 (5.6, 8.3) | 59 (51.5, 66.6) | 16 (7, 46) | 4.5 (1.6, 14.1) | 0.045 (0.030, 0.063) | 6.0 (3.8, 13.4) | 9.3 (7.8, 12.1) | 10.8 (6.4, 24.4) | 20.2 (15.4, 24.3) |
| TT | 7.4 (6.0, 9.0) | 59.8 (51.5, 67.6) | 21 (10, 43.5) | 5.8 (1.9, 14.2) | 0.056 (0.031, 0.084) | 8.32 (3.9, 15.1) | 10.5 (7.9, 11.9) | 12.8 (7.1, 36.2) | 16.6 (13.1, 21.2) |
| *P* values ^#^ | 0.245 | 0.872 | 0.034 | 0.181 | 0.409 | **0.010** | 0.156 | 0.144 | **0.016** |
| Post hoc multiple comparisons ^¶^ | | | | | | | | | |
| CC vs. CT | 0.951 | 0.726 | 0.046 | 0.151 | 0.997 | 0.023 | 0.125 | 0.214 | 0.535 |
| CC vs. TT | 0.104 | 0.847 | **0.013** | 0.085 | 0.203 | **0.006** | 0.077 | 0.051 | 0.024 |
| CT vs. TT | 0.141 | 0.633 | 0.431 | 0.567 | 0.231 | 0.196 | 0.518 | 0.330 | **0.005** |

FRI: Fracture related infection; WBC: white blood cell count; PMN%: percentage of polymorphonuclear; ESR: erythrocyte sedimentation rate; CRP: C-reactive protein; PCT: procalcitonin; IL-6: interleukin-6; TNF-α: tumor necrosis factor-α; SAA: serum amyloid A.

* These *P* values were obtained by the Mann-Whitney U test following comparisons between CC and CT genotypes, as only one sample of TT genotype was found of this SNV site.

^#^ These *P* values were obtained by the Kruskal-Wallis H test.

^^^ These *P* values were obtained by the Mann-Whitney U test.

^¶^ The alpha level was 0.017 for the post hoc multiple comparisons.
